# Analysis of the efficacy of the da Vinci robot in surgery for posterior mediastinal neurogenic tumors

**DOI:** 10.1186/s12893-022-01855-x

**Published:** 2022-12-06

**Authors:** Ziqiang Hong, Wenxi Gou, Baiqiang Cui, Yannan Sheng, Xiangdou Bai, Dacheng Jin, Yingjie Lu, Yunjiu Gou

**Affiliations:** 1 The First Clinical Medical College of Gansu University of Chinese Medicine, 35 East Dingxi Road, Lanzhou, 730000 Gansu China; 2 First Department of Thoracic Surgery, Gansu Provincial Hospital, 204 Donggang West Road, Lanzhou, 730000 Gansu China; 3 Southern Medical University, 1023-1063 Shatai South Road, Guangzhou, 510515 Guangdong China

**Keywords:** da Vinci Robotic Surgical System, Minimally invasive thoracic surgery, Mediastinal tumor, Neurogenic tumors

## Abstract

**Background:**

The present research is designed to evaluate the short-term outcome of robot-assisted thoracoscopic surgery (RATS) for the treatment of posterior mediastinal neurogenic tumors.

**Methods:**

We retrospectively analyzed clinical data on 39 consecutive patients with mediastinal neurogenic tumors after RATS treatment completed by the same operator in the Department of Thoracic Surgery, Gansu Provincial People's Hospital from January 2016 to September 2022. There were 22 males and 17 females with a mean age of (35.1 ± 6.9) years in this analysis report. The tumors of the patients were localized and evaluated preoperatively using magnetic resonance imaging (MRI) or enhanced CT.

**Results:**

All 39 patients successfully underwent the resection of posterior mediastinal neurogenic tumors under RATS, and no conversion to thoracotomy occurred during the operations. The average operative time was (62.1 ± 17.2) min, the average docking time was (10.1 ± 2.5) min, the average intraoperative bleeding was (32.8 ± 19.5) ml, the average 24-h postoperative chest drainage was (67.4 ± 27.9) ml, the average postoperative chest drainage time was (2.2 ± 1.3) days and the average post-operative hospital stay was (3.2 ± 1.3) days. Postoperative complications occurred in 3 patients, including 2 patients with transient Horner's syndrome after surgery and 1 patient with transient anhidrosis of the affected upper limb after surgery.

**Conclusion:**

RATS for posterior mediastinal neurogenic tumors is safe, effective, feasible and bring the superiority of robotic surgical system into full play.

## Background

Neurogenic tumors of the posterior mediastinum are one of the most common mediastinal tumors in adults, accounting for over 75% of posterior mediastinal tumors and 10–34% of all mediastinal tumors [1, 2]. The disease is mostly young and middle-aged patients, and 90% of patients are benign lesions [3]. At present, the specific causes of the diseased neurological tumors clinically have not completely studied thoroughly[3]. Traditional open thoracotomy for mediastinal tumors uses a posterior lateral chest incision to remove the ribs or enter the chest through the rib cage, which is a long and damaging incision [4]. In 1992, Landreneau et al. [5] reported a case of successful resection of a posterior mediastinal tumour by video-assisted thoracoscopic surgery (VATS), which laid the foundation for minimally invasive surgery in the resection of mediastinal tumors.

In recent years, the da Vinci robot has further demonstrated its advantages for complex movements in enclosed spaces thanks to features, such as its 3D field of view and flexible robotic arms [6, 7]. Its progressive development and importance in the field of surgery. Since the application of the da Vinci Robotic Surgical system in clinical surgery, the first case of a mediastinal neurogenic tumour after RATS resection was reported by Ruurda et al. [8] in 2003. However, there is still insufficient evidence of its effectiveness in the immediate postoperative period following resection of posterior mediastinal tumors. Since the introduction of the da Vinci Robotic Surgical System (da Vinci Si Surgical System) in our hospital in January 2016, 39 posterior superior mediastinal tumour operations have been performed, and we experience its significant advantages in the resection of neurogenic tumors in the posterior superior mediastinum, as reported below.

## Patients and methods

### Patients

In this single-centre retrospective study, we included and analysed 39 consecutive patients with posterior mediastinal neurogenic tumors treated with RATS completed by the same operator in the Department of Thoracic Surgery, Gansu Provincial People's Hospital from January 2016 to September 2022.

Inclusion criteria: (1) preoperative tumour localisation using MRI or enhanced CT, tumour size < 8 cm and resectable posterior mediastinal neurogenic tumour; (2) willingness to undergo robot-assisted surgery. Exclusion criteria: (1) anterior and middle mediastinal tumors; (2) patients with poor cardiopulmonary function or severe cardiac arrhythmias. For patients with intervertebral foraminal invasion, regular post-operative follow-up visits to the clinic are required to monitor for tumour recurrence.

### Operative methods

**Fig. 1:**
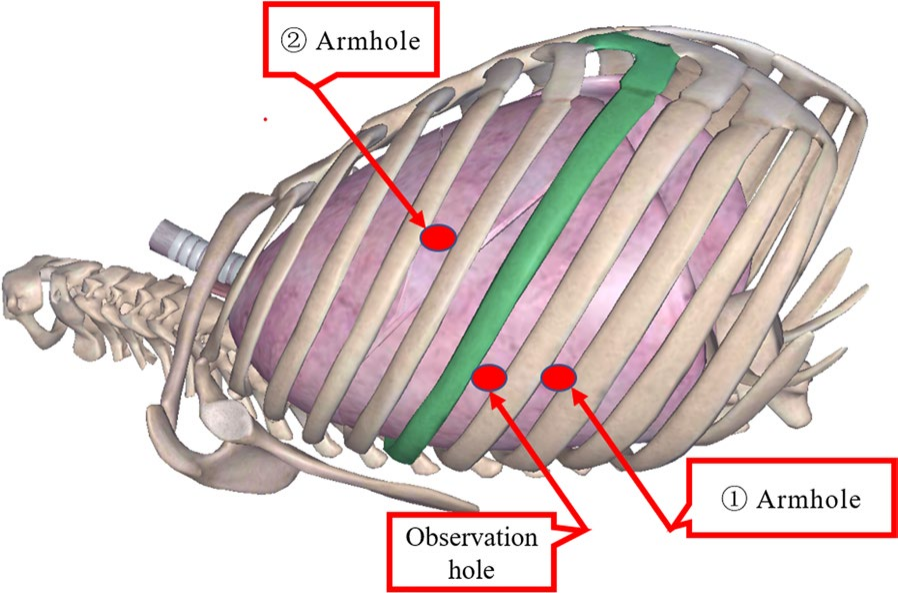
The "6-4-7" orifice approach for posterior superior mediastinal tumour

**Fig. 2:**
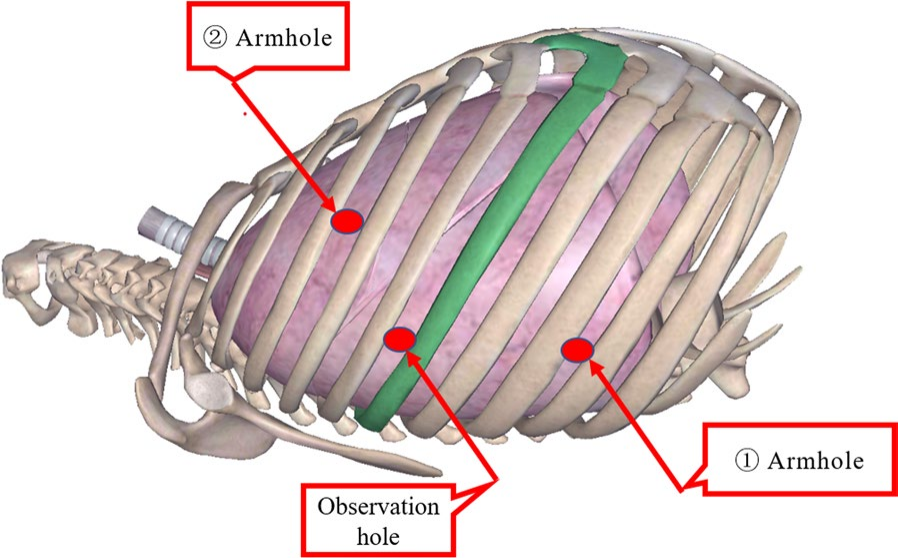
The "5-3-8" orifice approach for posterior inferior mediastinal tumour

**Fig. 3:**
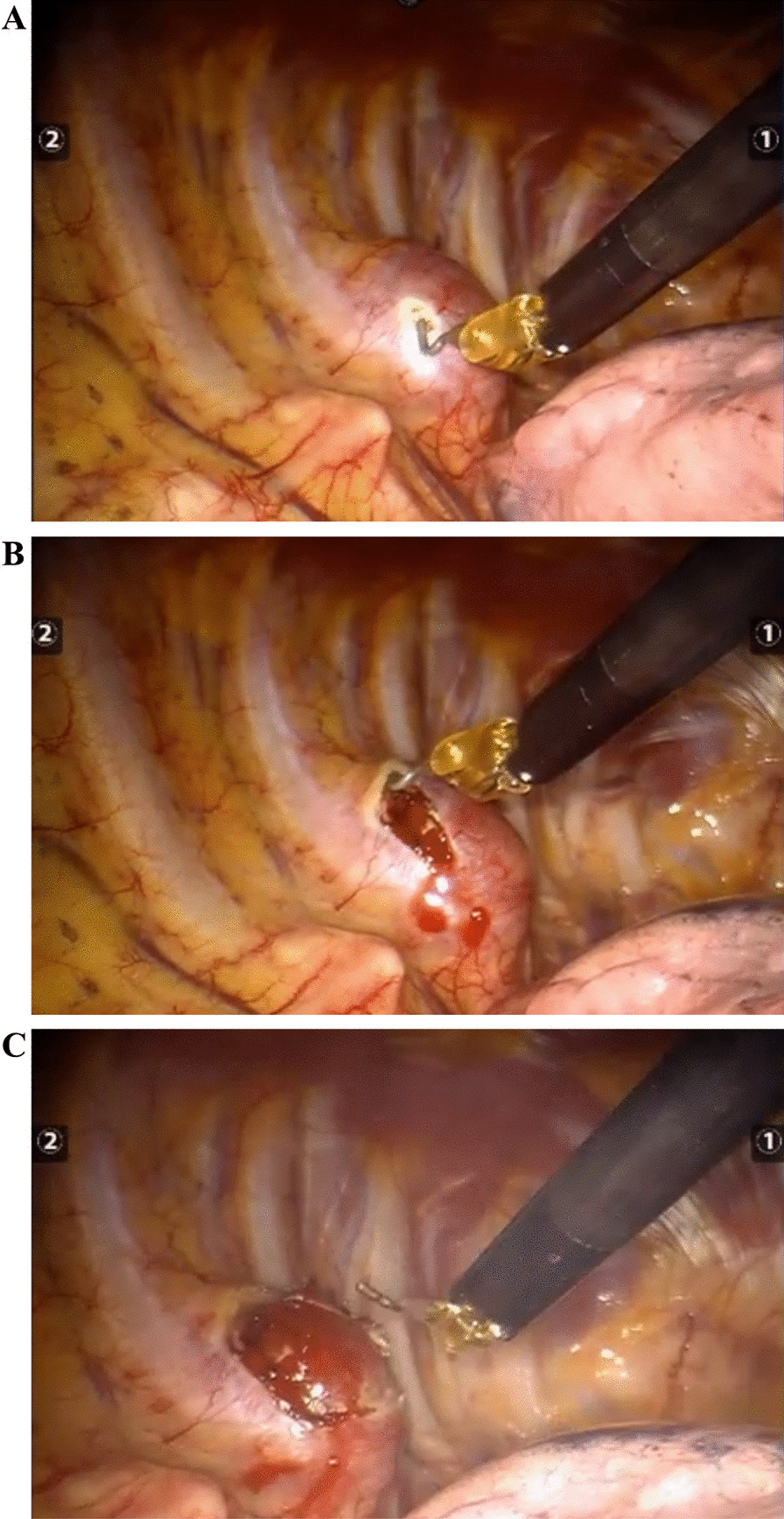
**A** Perform fine dissection of the tumor. **B** Perform fine dissection of the tumor. **C** Perform fine dissection of the tumor

**Fig. 4:**
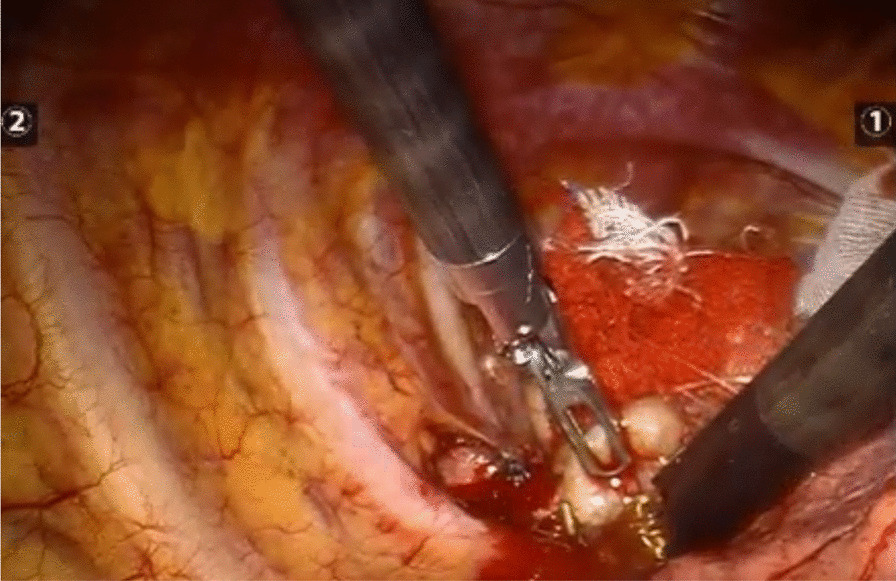
Removal of the tumor

**Fig. 5:**
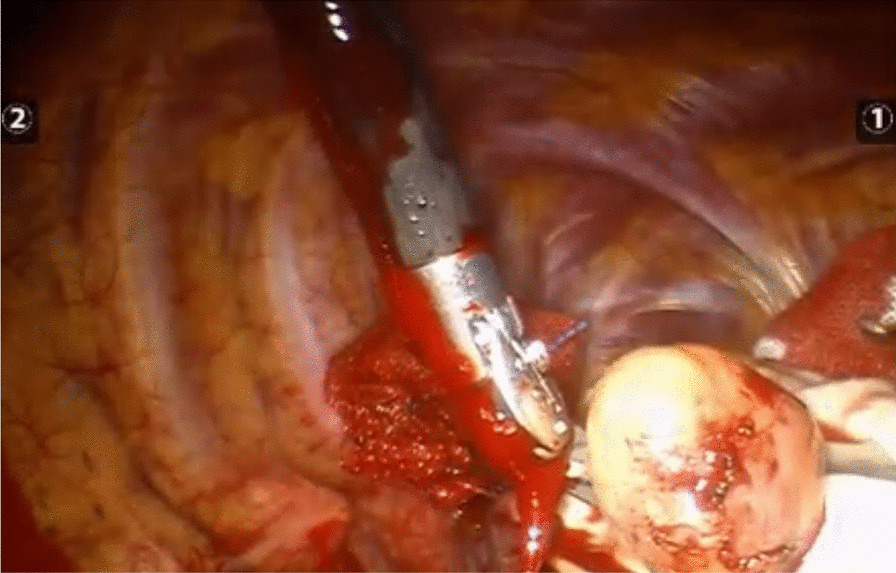
The excised tumor is placed on the specimen tape and removed

A three-hole, three-arm, full-port, CO_2_ artificial pneumothorax is used. Position: Lateral position, slightly leaning forward. Perforation location: Based on the CT image, a projection is outlined on the body surface and the perforation is designed according to this projection as required by the robot operated arm. For posterior superior mediastinum tumors, the hole is set up in the "6–4-7" method (Fig. 1) "6" is the observation hole, between the sixth intercostal space of the posterior axillary line on the affected side; "4" is the operation hole of the ② arm, between the fourth intercostal space of the anterior axillary line; "7" is the operation hole of the ① arm, between the seventh intercostal space of the posterior axillary line. For posterior inferior mediastinal tumors, the hole setting is the "5–3-8" method (Fig. 2) "5" is the observation hole, between the sixth intercostal space of the posterior axillary line on the affected side; "3" is the operation hole of the ② arm, between the fourth intercostal space of the anterior axillary line; "8" is the operation hole of the ① arm, between the seventh intercostal space of the posterior axillary line. Guiding the bedside instrument arm system in the direction of the inlet and the extension of the swelling line, connecting the robot system lens and the manipulator arm. The left arm is connected to the bipolar coagulation forceps and the right arm is connected to the unipolar coagulation hook. The tumour is separated sharply with the electrocoagulation hook, and the tumour is excised completely without an auxiliary port, and if there is bleeding or poor visualisation, an auxiliary hole is made between the ribs in front of the entrance port and a suction device is used to assist. Some of the intraoperative procedures are shown in Figs. 3A–C, 4 and 5.

### Data collection

Pre-operative data included basic clinical information such as patient's age, gender, body mass index (BMI), underlying disease, tumour size and type; intra-operative data included operative time, loading time and intra-operative bleeding; post-operative data included 24 h post-operative chest drainage, post-operative chest drainage time, post-operative length of stay and post-operative complications.

### Statistical analysis

SPSS 26.0 software(SPSS Inc., Chicago, IL, USA) was used for statistical analysis, and measurement data conforming to a normal distribution were described by mean ± standard deviation; measurement data not conforming to a normal distribution were described by median (upper and lower quartiles) [M(P25, P75)]; count data were described by frequency and percentage (%).

### Ethical review

This study has been reviewed by the Ethics Committee of Gansu Provincial People's Hospital, approval number: 2022-344. All patients signed the informed consent form for surgery before surgery.

## Results

### Patient general information

A total of 39 patients with resected mediastinal neurogenic tumors after RATS were included in this study, of whom 22 (56.4%) were male and 17 (43.6%) were female. The mean age of the patients was (35.1 ± 6.9) years, BMI was (23.1 ± 4.0) kg/m^2^ and tumour size was (4.3 ± 1.2) cm. There were 25 patients (64.1%) with no symptoms, 6 patients (15.3%) with chest pain, 4 patients (10.3%) with chest tightness and 4 patients (10.3%) with coughing symptoms. Among the tumour types, there were 23 cases (59.0%) of nerve sheath tumour, 13 cases (33.3%) of neurofibroma and 3 cases (7.7%) of ganglioneuroma; see Table 1.

**Table 1:** General information of patients [n = 39, mean ± standard deviation/case (%)

Clinical information	Data
Sex	
Male	22 (56.4)
Female	17 (43.6)
Age (years)	35.1 ± 6.9
BMI (kg/m^2^)	23.1 ± 4.0
Smoking history	10 (25.6)
Comorbidities	7 (17.9)
COPD	2 (5.1)
Hypertension	3 (7.7)
Diabetes	2 (5.1)
Tumour size (cm)	4.3 ± 1.2
Tumour type	
Nerve sheath tumors	23 (59.0)
Neurofibroma	13 (33.3)
Ganglioneuroma	3 (7.7)
Symptom	
No	25 (64.1)
Chest pain	6 (15.3)
Chest tightness	4 (10.3)
Cough	4 (10.3)

*BMI* body mass index, *COPD* chronic obstructive pulmonary disease

### Surgical results

The patient's surgical profile is detailed in Table 2. Surgery time: (62.1 ± 17.2) min; docking time: (10.1 ± 2.5) min; intraoperative bleeding: (32.8 ± 19.5) mL; postoperative 24 h chest drainage: (67.4 ± 27.9) mL; postoperative chest drainage time: (2.2 ± 1.3) days; postoperative hospital stay: (3.2 ± 1.3) days.

**Table 2:** Surgical data and postoperative complications of patients [n = 39, mean ± standard deviation/case (%)]

Clinical information	Data
Surgery time (min)	62.1 ± 17.2
Docking time (min)	10.1 ± 2.5
Blood loss (ml)	32.8 ± 19.5
Post-operative 24 h chest drainage (ml)	67.4 ± 27.9
Post-operative chest drainage time (d)	2.2 ± 1.3
Post-operative length of stay (d)	3.2 ± 1.3
Complications	3 (7.7)
Horner's Syndrome	2 (5.1)
No sweating on the affected upper limb	1 (2.6)

The procedure time is from cutting to sewing and does not include docking time

There were no serious surgical complications during the operation, such as intermediate open heart and perioperative death, and all patients recovered and were discharged after the operation. In terms of postoperative complications, there were two cases of postoperative transient Horner's syndrome and one case of transient absence of sweating on the affected upper limb; see Table 2.

## Discussion

Most posterior mediastinal tumors are of neurogenic origin and arise from the thoracic of neurological origin from the peripheral, sympathetic, and parasympathetic nerves within the tumors [9, 10]. Histologically, mediastinal tumors of neurogenic origin can be divided into nerve sheath tumors, sympathetic tumors (ganglion cell tumors account for about 40–60% of tumors), and neurofibromas. Currently, surgery is the primary treatment modality.

In traditional open-heart surgery for superior posterior mediastinal neurogenic tumors, obtaining a satisfactory surgical view of the tumour is difficult because of its location. Although thoracoscopic surgery may, to some extent, meet the satisfactory view requirements, obtaining high-quality surgical images with its 2D imaging system is challenging. The da Vinci Robot's naked-eye 3D imaging technology provides high-quality magnified images that can magnify the surgical field 10–15 times in three dimensions, allowing the operator to obtain a high-quality surgical image that facilitates the identification of the tumor's feeding vessels and its relationship with vital nerves [11]. The two ganglion cell tumors in this group were located at the entrance of the thorax at the top of the pleura. The upper part of the tumor was located deep in the neck. Thoracoscopic surgery is difficult to perform because open-heart surgery necessitates high stability and accuracy, which is only possible with a "semi-clam incision" [12].

Due to the high tumor position and small operating space of the tumor of the rear neuronal nerve source, the surgical field of vision is poor during routine opening surgery, the tumor leakage is difficult, and the movement of surgical instruments during thoracoscopy is often limited [13]. The difficulty of dissection is caused by losing accuracy. However, the accuracy of the robot surgery system is excellent. Some neurogenic tumors have the propensity to adhere to the surrounding tissue, making it more challenging to separate and ligate the feeding vessels of the tumour. For tumors originating from important motor nerves or close to important nerves, there is a high risk of nerve collateral damage due to the limited operating space and difficulty handling surgical instruments. The robotic arm of the da Vinci Robot Surgery System has seven free activities of wrist type, including advanced and retreating, wrist rotation, bending up, down, left and right directions, and the end-of-right grabbing movement [14]. The limited surgery field and fixed angle can be extended to include locations inaccessible to traditional equipment. These features of the da Vinci Robotic Surgical System allow it to operate not only in tight spaces but also to expose the surgical area more completely and provide a better view of difficult anatomical areas; thus, allowing surgeons to precisely isolate and treat blood vessels and vital nerves, improving surgical safety [15]. In this group, 30 tumors were located in the superior posterior mediastinum and were closely associated with the sympathetic chain. Nine ganglioneuroblastomas were located at the entrance to the thorax and originated from the sympathetic chain. However, for neurogenic tumors of the posterior mediastinum located at the top of the pleura, the removal of tumors within the capsule should be followed as far as possible. If the tumor and the capsule are too tightly adhered to retain the tumor capsule, the probability of damaging the peripheral nerves during the operation will increase. In this study, two patients developed transient Horner's syndrome after surgery, and one patient developed an absence of sweating in the affected upper extremity. All three patients had posterior mediastinal tumors in the pleural apex. There is a lower rate of postoperative complications than those reported by Endo et al. [16] and Cardillo et al. [17] for patients with posterior mediastinal tumors in the pleural apex treated with VATS. The strong adherence between the tumor and the capsule in pleural apex tumors will significantly increase the incidence of peripheral nerve injury. The stellate ganglion is usually close to the tumor; hence it is easier to damage the stellate ganglion during surgery, resulting in Horner's syndrome. In VATS, the pleural apex is difficult to visualize. In contrast, in RATS, the pleural apex is better visualized due to its 3D field of view and 10 × magnification images. There is less chance of damaging the nerve, so the postoperative complication rate is lower in RATS. Furthermore, in this study, the three postoperative complications occurred at the initial stage of RATS performed by the operator due to the lack of experience in resecting posterior mediastinal tumors in the pleural apex. Moreover, the complication rate decreased significantly at a later stage as the surgical experience and proficiency in RATS operation increased.

The da Vinci Robotic Surgical System requires only one 1.2 cm incision for access and two 0.8 cm incisions to manipulate the posterior mediastinal neurogenic tumour. The intraoperative use of an 8 mm Hg pneumothorax does not increase airway resistance but significantly increases intraoperative exposure. Usually, we do not use an auxiliary port. When the gauze is fed or when haemostatic powder needs to be sprayed on the wound, the instrument is withdrawn temporarily and is fed through the instrument hole. When the tumour needs to be removed after excision, one arm trocar is removed, and the endoscopic retriever is used to remove the specimen. We usually use an anterior chest wall incision to remove the specimen because of its wide rib space and thin muscle tissue. In the early stages, it takes longer to remove the specimen from the tiny single hole, and then it is easier to remove the specimen by extending the incision to the size of the tumour diameter.

The position of the body and the incision during robotic surgery determines the ease and success of the procedure. Usually, we choose the 6th intercostal space in the posterior axillary line as the observation hole position. When the tumour is in a high position, a lateral folding position is selected to prevent the lens arm from compressing the hip, and the instrument arm holes are chosen at the 4th intercostal space in the anterior axillary line and the 7th intercostal space in the posterior axillary line. Unlike lumpectomy or open surgery, it is not the case that the closer the incision is to the swelling, the greater the movement of the large arm when the tip of the instrument is moved laterally the same distance, causing more opportunities for collision and thus limiting the movement of the instrument. Therefore, choosing a more distant incision is more beneficial to the operation.

We use the bipolar electrocoagulation grasper with left hand and the electrocoagulation hook with right hand to complete the whole operations. The electrocoagulation hook is separated by layered electrocoagulation, and the bipolar grasper deals with the trophoblastic vessels and assists in exposure. However, because the tumour is usually brittle and does not have a tough outer membrane, and the grasper does not have force feedback, it cannot complete the "clamping" action during the freehand operation.

This study has some limitations and shortcomings. First, the results may be biased due to the single-center, small number of cases and retrospective study; second, the study lacks long-term survival analysis and further data refinement through follow-up is prepared.

## Conclusion

In summary, the da Vinci robotic posterior superior mediastinal neurogenic tumour resection is safe and feasible, with good surgical results. For the resection of neurogenic tumors in narrow spaces such as the pleural apex, the da Vinci Robotic Surgical System has distinct advantages, especially in preserving nerve function.

## Data Availability

The datasets used and/or analysed during the current study are available from the corresponding author on reasonable request.

## Abbreviations

RATS: Robot-assisted thoracoscopic surgery
MRI: Magnetic resonance imaging
VATS: Video-assisted thoracoscopic surgery
BMI: Body mass index
COPD: Chronic obstructive pulmonary disease
